# Methyl (*Z*)-3-({5-[(*E*)-(*tert*-butyl­amino)­methyl­idene]-4-oxo-4,5-dihydro-1,3-thia­zol-2-yl}sulfan­yl)prop-2-enoate

**DOI:** 10.1107/S1600536810030849

**Published:** 2010-08-18

**Authors:** Robabeh Baharfar, Nasim Porahmad, S. Mohammad Vahdat

**Affiliations:** a Department of Chemistry, University of Mazandaran, 47415 Babolsar, Iran

## Abstract

In the title compound, C_12_H_16_N_2_O_3_S_2_, the *S*-vinyl, and *tert*-butyl­enamine fragments make dihedral angles of 14.19 (2) and 0.85 (2)°, respectively, with the thia­zole ring. In the crystal, mol­ecules are linked into chains with graph-set motifs *C*(5) along [100] by C—H⋯O inter­actions. The mol­ecular conformation is stabilized by an intra­molecular N—H⋯O hydrogen bond.

## Related literature

The thia­zole ring system can be found in natural compounds such as thia­mine (Baia, *et al.*, 2008 ▶) and scleritodermin A (Wu & Yang, 2007 ▶). Thia­zole derivatives exhibit varied pharmaceutical properties including anti­cancer (Lesyk *et al.*, 2006 ▶, 2007 ▶), anti­convulsant (Siddiqui & Ahsan, 2010 ▶), anti­psychotic (Satoh *et al.*, 2009 ▶), anti­bacterial and anti­fungal (Abdel-Wahab *et al.*, 2009 ▶; Vijaya Raj *et al.*, 2007 ▶), anti­tubercular (Shiradkar, Murahari *et al.*, 2007 ▶), anti­microbial (Shiradkar, Kumar *et al.*, 2007 ▶), analgesic and anti-inflammatory (Koz’minykh *et al.*, 2004 ▶). For synthetic methods for thiazoles, see: Andrushko *et al.* (2001 ▶); Bourahla *et al.* (2007 ▶); Fakhari *et al.* (2008 ▶); Potikha *et al.* (2008 ▶). For hydrogen-bond motifs, see: Bernstein *et al.* (1995 ▶).
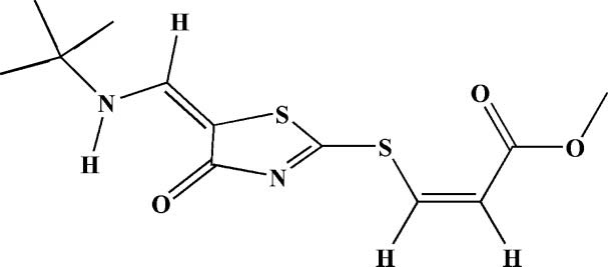


## Experimental

### 

#### Crystal data

C_12_H_16_N_2_O_3_S_2_
*M* *_r_* = 300.39
Monoclinic, 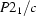

*a* = 6.011 (2) Å
*b* = 19.333 (7) Å
*c* = 12.870 (5) Å
β = 96.502 (8)°
*V* = 1485.9 (10) Å^3^
*Z* = 4
Mo *K*α radiation
μ = 0.36 mm^−1^
*T* = 120 K
0.20 × 0.10 × 0.10 mm

#### Data collection

Bruker SMART 1000 CCD area-detector diffractometer
Absorption correction: multi-scan (*SADABS*; Sheldrick, 1998 ▶) *T*_min_ = 0.951, *T*_max_ = 0.965
15867 measured reflections
3939 independent reflections
3125 reflections with *I* > 2σ(*I*)
*R*_int_ = 0.052

#### Refinement

*R*[*F* ^2^ > 2σ(*F* ^2^)] = 0.046
*wR*(*F* ^2^) = 0.108
*S* = 1.00
3939 reflections
177 parameters
H-atom parameters constrained
Δρ_max_ = 0.66 e Å^−3^
Δρ_min_ = −0.28 e Å^−3^

### 

Data collection: *SMART* (Bruker, 1998 ▶); cell refinement: *SAINT-Plus* (Bruker, 1998 ▶); data reduction: *SAINT-Plus*; program(s) used to solve structure: *SHELXTL* (Sheldrick, 2008 ▶); program(s) used to refine structure: *SHELXTL*; molecular graphics: *SHELXTL*; software used to prepare material for publication: *SHELXTL*.

## Supplementary Material

Crystal structure: contains datablocks I, global. DOI: 10.1107/S1600536810030849/bx2284sup1.cif

Structure factors: contains datablocks I. DOI: 10.1107/S1600536810030849/bx2284Isup2.hkl

Additional supplementary materials:  crystallographic information; 3D view; checkCIF report

## Figures and Tables

**Table 1:** Hydrogen-bond geometry (Å, °)

*D*—H⋯*A*	*D*—H	H⋯*A*	*D*⋯*A*	*D*—H⋯*A*
N2—H2*N*⋯O1	0.90	2.21	2.777 (2)	120
C8—H8*A*⋯O1^i^	0.95	2.18	3.117 (3)	171

Symmetry code: (i) 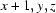.

**Fig. 1.**
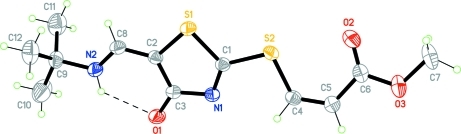
The molecular structure of the title compound, showing 50% probability displacement ellipsoids and the atomic numbering. The dashed lines show N—H··· O intramolecular interaction.

**Fig. 2.**
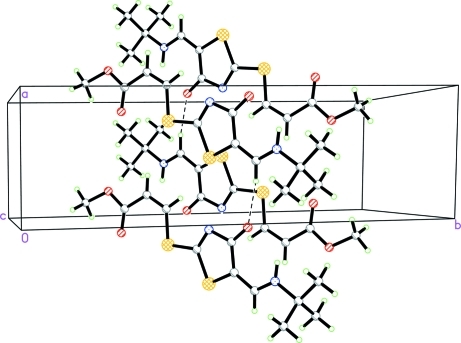
The crystal packing of the title compound viewed down the c-axis showing linking of molecules along the a-axis by the intermolecular C—H···O hydrogen bonds. The dashed lines show intermolecular interactions.

## supplementary crystallographic information

### Comment

The thiazole ring system can be fined in natural compounds like thiamine (vitamin B_1_) (Baia, *et al.*, 2008), bistratamide H, archazolid A & B, siomycin A, didmolamide A, scleritodermin A, etc. (Wu & Yang, 2007). Thiazole derivatives exhibit different pharmaceutical properties, among them are: anticancer (Lesyk *et al.*, 2007; & Lesyk *et al.*, 2006), anticonvulsant (Siddiqui & Ahsan, 2010), antipsychotic-like (Satoh *et al.*, 2009), antibacterial, antifungal (Abdel-Wahab *et al.*, 2009; & Vijaya Raj *et al.*, 2007), antitubercular (Shiradkar, Murahari *et al.*, 2007), antimicrobial (Shiradkar, Kumar *et al.*, 2007), analgesic and anti-inflammatory (Koz'minykh *et al.*, 2004) activities. These compounds have been synthesized using different methods (Andrushko *et al.*, 2001; & Bourahla *et al.*, 2007; & Fakhari *et al.*, 2008; & Potikha *et al.*, 2008). We have succeeded in synthesizing a thiazole derivative using a three step reaction.  methods for theirWe report here the synthesis and crystal structure of the title compound (I). The molecular structure of (I) is illustrated in Fig 1. The fragments *S*-vinyl, and *tert*-butyl enamine makes angles of 14.19 (2) and 0.85 (2)° with the thiazole ring. In the crystal the molecules are linked into chains along [100] direction with graph-set notation C(5) motifs by a C—H···O interaction, (Bernstein, *et al.*, 1995) Fig. 2. The molecular conformation is stabilized by two intramolecular N—H···O and C—H···O hydrogen bonds. Z-configuration was assigned to the geometry of *S*-vinyl system on the basis of torsion angle of -1.86 (4)° between atom S_2_ and methoxy carbonyl group.

### Experimental

To a magnetically stirred solution of rhodanine (0.27 g, 2 mmol) and methyl acetylenecarboxylate (0.17 g, 2 mmol) in 10 ml CH~2~Cl~2~, was added dropwise over 10 min, tert-butyl isocyanide (0.45 g, 2 mmol) in 2 ml CH_2_Cl_2_ . The mixture was then refluxed for 24 h. The solvent was removed under pressure and the residue was purified by silica gel (Merck 230-400 mesh) column chromatography using n-hexane-diethyl ether (2:3) as eluent. Three products were isolated. The single crystals of the title compound were obtained from the n-hexane-ethyl acetate solution. Orange powder, yield 20%. ^1^H NMR (300 MHz, CDCl_3_): δ = 1.39 (9H, s, CMe_3_), 3.78 (3H, s, OMe), 6.12 (1H, d, ^3^JHH= 10.0 Hz, S—CH=CH), 7.52 (1H, d, ^3^JHH = 13.4 Hz, NH—CH=C), 8.41 (1H, d, ^3^JHH = 10.0 Hz, S—CH=CH), 10.12 (1H, d, ^3^JHH = 13.4 Hz, NH—CH=C). ^13^CNMR (75.5 MHz, CDCl_3_): δ= 29.94 (CMe_3_), 51.91 (OCH_3_), 53.98 (CMe_3_), 96.52 (NH— CH=C), 115.38 (CH=CH—C=O), 139.29 (CH=CH—C=O), 145.10 (NH—CH=C), 166.86 (C=N), 177.42 and 179.48 (2 C=O). IR (KBr) (ν/cm^-1^): 3313-3562 (NH), 1699 and 1643 (2 C=O), 1578 (C=N).

### Refinement

The hydrogen atom of NH group was found in difference Fourier synthesis. The H(C) atom positions were calculated. All hydrogen atoms were refined in isotropic approximation in riding model with with the Uiso(H) parameters equal to 1.2 Ueq(Ci), for methyl groups equal to 1.5 Ueq(Cii), where U(Ci) and U(Cii) are respectively the equivalent thermal parameters of the carbon atoms to which corresponding H atoms are bonded.

### Crystal data

**Table:**

C_12_H_16_N_2_O_3_S_2_	*F*(000) = 632
*M**_r_* = 300.39	*D*_x_ = 1.343 Mg m^−^^3^
Monoclinic, *P*2_1_/*c*	Mo *K*α radiation, λ = 0.71073 Å
Hall symbol: -P 2ybc	Cell parameters from 1769 reflections
*a* = 6.011 (2) Å	θ = 2–25°
*b* = 19.333 (7) Å	µ = 0.36 mm^−^^1^
*c* = 12.870 (5) Å	*T* = 120 K
β = 96.502 (8)°	Prism, orange
*V* = 1485.9 (10) Å^3^	0.20 × 0.10 × 0.10 mm
*Z* = 4	

### Data collection

**Table:**

Bruker SMART 1000 CCD area-detector diffractometer	3939 independent reflections
Radiation source: fine-focus sealed tube	3125 reflections with *I* > 2σ(*I*)
graphite	*R*_int_ = 0.052
phi and ω scans	θ_max_ = 29.0°, θ_min_ = 1.9°
Absorption correction: multi-scan (*SADABS*; Sheldrick, 1998)	*h* = −8→8
*T*_min_ = 0.951, *T*_max_ = 0.965	*k* = −26→26
15867 measured reflections	*l* = −17→17

### Refinement

**Table:**

Refinement on *F*^2^	Primary atom site location: structure-invariant direct methods
Least-squares matrix: full	Secondary atom site location: difference Fourier map
*R*[*F*^2^ > 2σ(*F*^2^)] = 0.046	Hydrogen site location: inferred from neighbouring sites
*wR*(*F*^2^) = 0.108	H-atom parameters constrained
*S* = 1.00	*w* = 1/[σ^2^(*F*_o_^2^) + (0.010*P*)^2^ + 1.980*P*] where *P* = (*F*_o_^2^ + 2*F*_c_^2^)/3
3939 reflections	(Δ/σ)_max_ = 0.001
177 parameters	Δρ_max_ = 0.66 e Å^−^^3^
0 restraints	Δρ_min_ = −0.28 e Å^−^^3^

### Special details

**Table:**

Geometry. All esds (except the esd in the dihedral angle between two l.s. planes) are estimated using the full covariance matrix. The cell esds are taken into account individually in the estimation of esds in distances, angles and torsion angles; correlations between esds in cell parameters are only used when they are defined by crystal symmetry. An approximate (isotropic) treatment of cell esds is used for estimating esds involving l.s. planes.
Refinement. Refinement of *F*^2^ against ALL reflections. The weighted *R*-factor wR and goodness of fit *S* are based on *F*^2^, conventional *R*-factors *R* are based on *F*, with *F* set to zero for negative *F*^2^. The threshold expression of *F*^2^ > σ(*F*^2^) is used only for calculating *R*-factors(gt) etc. and is not relevant to the choice of reflections for refinement. *R*-factors based on *F*^2^ are statistically about twice as large as those based on *F*, and *R*- factors based on ALL data will be even larger.

### Fractional atomic coordinates and isotropic or equivalent isotropic displacement parameters (Å^2^)

**Table:**

	*x*	*y*	*z*	*U*_iso_*/*U*_eq_	
S1	0.50629 (8)	0.53451 (3)	0.63467 (4)	0.03196 (13)	
S2	0.20485 (8)	0.62005 (3)	0.49185 (4)	0.03050 (13)	
O1	0.0477 (2)	0.46226 (9)	0.78666 (12)	0.0376 (4)	
O2	0.1152 (3)	0.72490 (9)	0.34743 (13)	0.0459 (4)	
O3	−0.2299 (3)	0.75225 (9)	0.27204 (13)	0.0438 (4)	
N1	0.0743 (3)	0.53679 (9)	0.64593 (13)	0.0282 (3)	
N2	0.4543 (3)	0.40971 (9)	0.87697 (14)	0.0318 (4)	
H2N	0.3101	0.4070	0.8892	0.038*	
C1	0.2330 (3)	0.56032 (10)	0.59558 (15)	0.0258 (4)	
C2	0.4049 (3)	0.48646 (10)	0.73331 (15)	0.0269 (4)	
C3	0.1648 (3)	0.49260 (10)	0.72696 (15)	0.0272 (4)	
C4	−0.0858 (3)	0.62176 (10)	0.46463 (15)	0.0276 (4)	
H4A	−0.1700	0.5904	0.5014	0.033*	
C5	−0.1981 (4)	0.66419 (10)	0.39564 (16)	0.0305 (4)	
H5A	−0.3567	0.6608	0.3840	0.037*	
C6	−0.0849 (4)	0.71564 (11)	0.33756 (16)	0.0324 (4)	
C7	−0.1334 (6)	0.80731 (14)	0.2156 (2)	0.0577 (7)	
H7A	−0.2472	0.8259	0.1624	0.087*	
H7B	−0.0076	0.7891	0.1817	0.087*	
H7C	−0.0800	0.8442	0.2645	0.087*	
C8	0.5374 (3)	0.44672 (10)	0.80461 (16)	0.0294 (4)	
H8A	0.6945	0.4460	0.8014	0.035*	
C9	0.5922 (4)	0.36708 (12)	0.95625 (17)	0.0361 (5)	
C10	0.4347 (5)	0.3335 (2)	1.0225 (3)	0.0814 (12)	
H10A	0.3303	0.3038	0.9788	0.122*	
H10B	0.3505	0.3692	1.0555	0.122*	
H10C	0.5197	0.3055	1.0767	0.122*	
C11	0.7586 (5)	0.41439 (16)	1.0220 (2)	0.0563 (7)	
H11A	0.6761	0.4501	1.0558	0.084*	
H11B	0.8583	0.4364	0.9766	0.084*	
H11C	0.8475	0.3869	1.0756	0.084*	
C12	0.7262 (5)	0.31403 (14)	0.9007 (2)	0.0548 (7)	
H12A	0.6231	0.2844	0.8562	0.082*	
H12B	0.8157	0.2856	0.9529	0.082*	
H12C	0.8258	0.3382	0.8575	0.082*	

### Atomic displacement parameters (Å^2^)

**Table:**

	*U*^11^	*U*^22^	*U*^33^	*U*^12^	*U*^13^	*U*^23^
S1	0.0213 (2)	0.0381 (3)	0.0374 (3)	0.00031 (19)	0.00743 (19)	0.0062 (2)
S2	0.0267 (2)	0.0302 (2)	0.0356 (3)	−0.00246 (19)	0.00762 (19)	0.0066 (2)
O1	0.0256 (7)	0.0497 (9)	0.0387 (8)	−0.0037 (6)	0.0087 (6)	0.0120 (7)
O2	0.0472 (10)	0.0462 (10)	0.0439 (9)	−0.0114 (8)	0.0037 (7)	0.0139 (8)
O3	0.0543 (10)	0.0398 (9)	0.0380 (9)	0.0094 (8)	0.0081 (7)	0.0129 (7)
N1	0.0239 (8)	0.0303 (8)	0.0313 (8)	−0.0003 (6)	0.0068 (6)	0.0025 (7)
N2	0.0248 (8)	0.0362 (9)	0.0348 (9)	0.0008 (7)	0.0058 (7)	0.0050 (7)
C1	0.0234 (9)	0.0245 (9)	0.0296 (9)	−0.0002 (7)	0.0044 (7)	−0.0018 (7)
C2	0.0253 (9)	0.0271 (9)	0.0289 (10)	−0.0021 (7)	0.0061 (7)	0.0007 (7)
C3	0.0249 (9)	0.0284 (9)	0.0285 (9)	−0.0022 (7)	0.0043 (7)	−0.0007 (8)
C4	0.0274 (9)	0.0264 (9)	0.0300 (10)	−0.0016 (7)	0.0081 (7)	−0.0017 (7)
C5	0.0324 (10)	0.0292 (10)	0.0302 (10)	0.0011 (8)	0.0049 (8)	−0.0019 (8)
C6	0.0431 (12)	0.0279 (10)	0.0264 (10)	0.0009 (9)	0.0043 (8)	−0.0012 (8)
C7	0.080 (2)	0.0452 (14)	0.0496 (15)	0.0083 (14)	0.0150 (14)	0.0224 (12)
C8	0.0236 (9)	0.0312 (10)	0.0339 (10)	−0.0012 (7)	0.0049 (7)	−0.0029 (8)
C9	0.0378 (11)	0.0367 (11)	0.0332 (11)	0.0052 (9)	0.0018 (9)	0.0067 (9)
C10	0.0503 (17)	0.109 (3)	0.087 (2)	0.0109 (18)	0.0177 (16)	0.059 (2)
C11	0.0666 (18)	0.0566 (17)	0.0426 (14)	0.0052 (14)	−0.0073 (13)	−0.0017 (12)
C12	0.0664 (18)	0.0447 (14)	0.0521 (16)	0.0155 (13)	0.0012 (13)	0.0014 (12)

### Geometric parameters (Å, °)

**Table:**

S1—C1	1.736 (2)	C5—H5A	0.9500
S1—C2	1.738 (2)	C7—H7A	0.9800
S2—C4	1.743 (2)	C7—H7B	0.9800
S2—C1	1.759 (2)	C7—H7C	0.9800
O1—C3	1.246 (2)	C8—H8A	0.9500
O2—C6	1.209 (3)	C9—C10	1.493 (4)
O3—C6	1.343 (3)	C9—C12	1.530 (3)
O3—C7	1.447 (3)	C9—C11	1.537 (4)
N1—C1	1.295 (2)	C10—H10A	0.9800
N1—C3	1.409 (3)	C10—H10B	0.9800
N2—C8	1.317 (3)	C10—H10C	0.9800
N2—C9	1.488 (3)	C11—H11A	0.9800
N2—H2N	0.9000	C11—H11B	0.9800
C2—C8	1.379 (3)	C11—H11C	0.9800
C2—C3	1.441 (3)	C12—H12A	0.9800
C4—C5	1.335 (3)	C12—H12B	0.9800
C4—H4A	0.9500	C12—H12C	0.9800
C5—C6	1.458 (3)		
			
C1—S1—C2	88.09 (10)	H7A—C7—H7C	109.5
C4—S2—C1	99.97 (9)	H7B—C7—H7C	109.5
C6—O3—C7	115.7 (2)	N2—C8—C2	122.48 (19)
C1—N1—C3	109.79 (16)	N2—C8—H8A	118.8
C8—N2—C9	124.02 (18)	C2—C8—H8A	118.8
C8—N2—H2N	127.3	N2—C9—C10	107.0 (2)
C9—N2—H2N	108.6	N2—C9—C12	109.43 (19)
N1—C1—S1	118.72 (15)	C10—C9—C12	112.1 (3)
N1—C1—S2	126.69 (15)	N2—C9—C11	108.98 (19)
S1—C1—S2	114.53 (11)	C10—C9—C11	111.1 (2)
C8—C2—C3	125.64 (18)	C12—C9—C11	108.2 (2)
C8—C2—S1	124.06 (15)	C9—C10—H10A	109.5
C3—C2—S1	110.26 (14)	C9—C10—H10B	109.5
O1—C3—N1	122.95 (18)	H10A—C10—H10B	109.5
O1—C3—C2	123.95 (18)	C9—C10—H10C	109.5
N1—C3—C2	113.10 (16)	H10A—C10—H10C	109.5
C5—C4—S2	124.43 (16)	H10B—C10—H10C	109.5
C5—C4—H4A	117.8	C9—C11—H11A	109.5
S2—C4—H4A	117.8	C9—C11—H11B	109.5
C4—C5—C6	122.0 (2)	H11A—C11—H11B	109.5
C4—C5—H5A	119.0	C9—C11—H11C	109.5
C6—C5—H5A	119.0	H11A—C11—H11C	109.5
O2—C6—O3	123.7 (2)	H11B—C11—H11C	109.5
O2—C6—C5	124.3 (2)	C9—C12—H12A	109.5
O3—C6—C5	111.96 (19)	C9—C12—H12B	109.5
O3—C7—H7A	109.5	H12A—C12—H12B	109.5
O3—C7—H7B	109.5	C9—C12—H12C	109.5
H7A—C7—H7B	109.5	H12A—C12—H12C	109.5
O3—C7—H7C	109.5	H12B—C12—H12C	109.5
			
C3—N1—C1—S1	0.2 (2)	S1—C2—C3—N1	−1.8 (2)
C3—N1—C1—S2	−177.08 (15)	C1—S2—C4—C5	174.49 (18)
C2—S1—C1—N1	−1.00 (17)	S2—C4—C5—C6	−1.9 (3)
C2—S1—C1—S2	176.56 (12)	C7—O3—C6—O2	−2.9 (3)
C4—S2—C1—N1	−11.2 (2)	C7—O3—C6—C5	176.5 (2)
C4—S2—C1—S1	171.44 (11)	C4—C5—C6—O2	−1.0 (3)
C1—S1—C2—C8	179.57 (19)	C4—C5—C6—O3	179.54 (19)
C1—S1—C2—C3	1.47 (15)	C9—N2—C8—C2	−179.01 (19)
C1—N1—C3—O1	−179.05 (19)	C3—C2—C8—N2	−0.7 (3)
C1—N1—C3—C2	1.0 (2)	S1—C2—C8—N2	−178.46 (16)
C8—C2—C3—O1	0.3 (3)	C8—N2—C9—C10	−179.7 (3)
S1—C2—C3—O1	178.34 (17)	C8—N2—C9—C12	−58.0 (3)
C8—C2—C3—N1	−179.81 (19)	C8—N2—C9—C11	60.1 (3)

### Hydrogen-bond geometry (Å, °)

**Table:**

*D*—H···*A*	*D*—H	H···*A*	*D*···*A*	*D*—H···*A*
N2—H2N···O1	0.90	2.21	2.777 (2)	120
C4—H4A···N1	0.95	2.46	2.926 (3)	110
C8—H8A···O1^i^	0.95	2.18	3.117 (3)	171

Symmetry codes:  (i) *x*+1, *y*, *z*.
